# Barriers to alcohol and other drug treatment use among Black African and Coloured South Africans

**DOI:** 10.1186/1472-6963-13-177

**Published:** 2013-05-17

**Authors:** Bronwyn Myers

**Affiliations:** 1 Alcohol and Drug Abuse Research Unit, South African Medical Research Council, PO Box 19070, Tygerberg, 7505, South Africa; 2 Department of Psychiatry and Mental Health, University of Cape Town, Cape Town, South Africa

**Keywords:** Alcohol and other drug treatment, Racial disparities, Barriers to treatment, South Africa

## Abstract

**Background:**

There are racial disparities in the use of alcohol and other drug (AOD) treatment services in South Africa but little is known about the factors contributing to these disparities. This study aimed to redress this gap through identifying differences in barriers to AOD treatment use among Black African and Coloured persons from Cape Town, South Africa. The Behavioral Model of Health Services Utilization was used as an analytic framework.

**Methods:**

A case-control design was used to compare 434 individuals with AOD problems who had accessed treatment with 555 controls who had not accessed treatment on a range of variables. Logistic regression procedures were employed to examine the unique profile of variables associated with treatment utilization for Black African and Coloured participants.

**Results:**

After controlling for the influence of treatment need and predisposing factors on treatment use, several barriers to treatment were identified. Greater awareness of treatment options and fewer geographic access and affordability barriers were strongly associated with an increased likelihood of AOD treatment use for both race groups. However, Black African persons were more vulnerable to the effects of awareness and geographic access barriers on treatment use. Stigma consciousness was only associated with AOD treatment utilization for Coloured participants.

**Conclusion:**

Differences in barriers to AOD treatment use were found among Black African and Coloured South Africans. Targeted interventions that address the unique profile of barriers experienced by each race group are needed to improve AOD treatment use by these underserved groups. Several strategies for improving the likelihood of treatment entry are suggested.

## Background

Findings from national epidemiological research point to high rates of untreated alcohol and other drug (AOD) use disorders in South Africa [1]. This is cause for concern as AOD use poses a significant threat to public health in the country. These problems are particularly prevalent in the Western Cape province, with a recent representative survey reporting significantly higher rates for AOD use disorders (20.3%) in this province compared to the national average of 13.3% [1]. Cape Town, the capital of the Western Cape, is particularly affected by AOD-related problems with this city reporting the highest proportion of alcohol and drug positive arrestees [2] and emergency room patients [3] compared to other major cities in the country. Taken together, these findings highlight the need for accessible AOD treatment services in Cape Town.

Despite the demand for AOD treatment in this region, access to treatment is limited in South Africa [4]. While the limited availability of AOD services restricts access to treatment for all South Africans, treatment is relatively more difficult to access for people from Black African and Coloured (that is people of mixed race ancestry who form a unique cultural group) communities disadvantaged during the course of apartheid who remain under-represented in AOD treatment facilities [5]. These racial disparities in access to treatment are probably an artifact of the apartheid system of governance. During apartheid, race was a major determinant of access to health and social services (including AOD treatment), with Whites having more access to public services than Coloured or Black African South Africans [6,7]. These disparities arose from the legislated geographic segregation of race groups and the distribution of resources along racial lines. This geographical apartheid forced Black African and Coloured South Africans to live in township areas with limited infrastructure that were located considerable distances from the well-resourced urban areas reserved for the use of White South Africans [6].

Despite 18 years of democracy, South Africa is still grappling with the legacy of apartheid and the challenges of promoting equitable access to public services for all racially-defined social groups. Race remains an important marker of socio-economic advantage in the country which impacts on the extent to which individuals are able to access services [7]. Poor Black African and Coloured persons continue to experience the most difficulty in accessing health services (including AOD services) relative to other groups [6,8]. Only about 16% of South Africans are members of private health insurance schemes (known as medical schemes) and use health services in the private health sector. The remaining 84% of the population, disproportionately represented by poor Black African and Coloured South Africans, are mainly dependent on the overburdened and under-resourced public services sector for access to health care (although some pay out-of-pocket for basic primary care services in the private sector) [8]. Racial disparities in access to AOD treatment are likely to be entrenched by the limited availability of free AOD treatment services in the public service sector. For example in the Western Cape province, which arguably is among the better resourced provinces in terms of access to AOD treatment services [4,5], there are only three AOD outpatient services and three inpatient facilities available in the public service sector that offer free treatment services. The remainder of the AOD inpatient treatment facilities in the province are either private non-profit facilities that offer reduced-cost services but still charge co-payment fees or private for-profit facilities that cater for the proportion of the population with access to medical insurance and charge high fees. Apart from outpatient services offered in the public sector, there are also outpatient services provided by private non-profit treatment providers. Although these agencies provide low-cost services, some do require clients to make a financial contribution towards each appointment. While these AOD services are among the least expensive, these are often unaffordable to poor South Africans, especially when coupled with the costs of travelling to these services.

However, there has been no research on affordability barriers and other factors that may contribute to racial disparities in AOD treatment use. This is worrisome as understanding how barriers and facilitators to AOD treatment entry vary across racial groups is a prerequisite for developing targeted interventions aimed at expanding AOD treatment coverage for underserved groups. This study aimed to redress this gap by identifying differences in barriers and facilitators to AOD treatment use among Black African and Coloured persons from Cape Town, South Africa.

The theoretical basis for this study was the Behavioral Model of Health Services Utilization (BHSU) [9]. This model was selected because it has been used extensively to examine behavioral health services use, including the use of AOD services [9-11] and also because it explicitly recognises that need for care and psychological and social factors influence access to treatment; factors that are often downplayed in other models of access [11]. This model adopts a systems approach that integrates a range of individual, contextual and provider variables associated with health services use into a single framework. It allows researchers to examine why individuals use health services, measure equitable access to health services, and guide policy development concerning service use. The model is thought to both predict and explain health service utilisation. Specifically, the BHSU suggests that health service use is a function of the separate and combined influence of predisposing characteristics, factors that enable or restrict health service use, and need variables. Predisposing characteristics (such as demographic and attitudinal-belief variables) exist within a person prior to the onset of a specific health need and predispose a person to use services. Enabling factors represent the person’s actual ability to obtain health services and include affordability factors, geographic accessibility and awareness of services, as well as psychological functioning. Service need variables reflect internal and external perceptions that health problems are severe enough to warrant the use of services [9,10].

## Methods

### Study design

This study used a case-control design to compare cases and controls on a range of variables thought to be associated with AOD treatment utilization. Cases were defined as persons from disadvantaged communities with AOD problems who reported AOD treatment use in the 12 months preceding the study. Controls were defined as persons from disadvantaged communities who had not used AOD treatment prior to this study, despite having AOD problems that required treatment. To control for selection bias, frequency matching techniques were used to match cases and controls on gender and race dimensions. To limit recall bias, we used time-line follow back (TLFB) procedures to collect retrospective data [12].

### Recruitment and data collection procedures

Convenience samples of Black African and Coloured people were recruited from treatment programmes and communities using snowball sampling techniques.

#### Recruitment of cases

Cases were identified at AOD treatment facilities in Cape Town, which served as starting points for sampling. To be selected for inclusion in the study, potential cases had to be at least 18 years old, self-identify as Black African or Coloured, earn less than ZAR 2500 per month (at the time of the study 1 USD = ZAR 9), have received treatment for AOD problems, and provide informed consent to participate in the study. Of the 440 persons screened for potential eligibility, all met the inclusion criteria. Having established eligibility, informed consent was obtained from potential cases to administer a questionnaire on access to treatment. As only six potential cases refused to participate in the study, the final sample comprised 434 cases. Fieldworkers then contacted these recruits to arrange a date and time for completion of the interviewer-administered questionnaire which took about 90 minutes to complete. Fieldworkers provided participants with refreshments, feedback, and referrals to services where requested.

#### Recruitment of controls

Controls were recruited by a team of experienced fieldworkers. To ensure controls represented the population of persons with AOD problems in disadvantaged communities, subjects were recruited from a range of these communities. Two residential areas from each of the six sub-structures of the Cape Town metropole were selected as key focus areas for sampling. To be selected as an area for sampling, the community had to consistently appear in the South African Community Epidemiology Network on Drug Use’s list of top ten residential areas for AOD problems [5], have been classified as a “Black African” or “Coloured” residential area under the apartheid regime, have high levels of health and social problems (defined as having a high prevalence of infectious diseases such as HIV and tuberculosis and a high prevalence of crime and violence), and be a low-income area.

Fieldworkers entered these communities by contacting organisations, leaders, and individuals with known interests in the AOD field and asking them to identify potential recruits to act as starting points for snowball sampling. Fieldworkers then screened these potential recruits for eligibility. To be selected as a control, potential recruits had to be at least 18 years old, self-identify as Black African or Coloured, earn less than ZAR2500 per month, have untreated AOD problems in need of treatment (assessed using the Texas Christian University (TCU) Drug Screen [13]), and provide informed consent to participate in the study. Of the 559 potential recruits screened, only four did not meet the study’s eligibility criteria. An overall response rate of 98.3% was obtained (N = 555). For eligible participants, fieldworkers obtained consent to administer a questionnaire on access to treatment which took approximately 90 minutes to complete. Fieldworkers provided participants with refreshments, feedback, and referrals to AOD services where requested. Ethical approval for this study was granted by the Ethics Review Board of the Faculty of Humanities at the University of Cape Town.

### Measures

#### TCU drug screen

The TCU Drug Screen-II [13] was used to screen potential controls for drug use severity and dependence. This screener has been used with treatment-seeking populations and community samples to screen for drug use severity and dependence [13]. The first nine items of the TCUDS are used to compute a continuous composite score that measures drug use severity [14]. Composite scores range from 0 to 9, with a composite score of three or more indicating relatively severe drug-related problems that correspond to a DSM-IV-TR drug dependence diagnosis [15] and indicating an objective need for treatment. The scale's overall reliability is good (α = .89) [13] and has good test-retest reliability (*r* = .97) [14]. This study obtained a Cronbach coefficient alpha of .88 for the scale. In addition, all controls scored above the cut-off point of 3, indicating an objective need for treatment.

#### Measures contained in the access to treatment questionnaire

This questionnaire, designed for South African populations, collected self-report information from various domains including demographics and social characteristics, AOD use and need for treatment, and barriers and facilitators to AOD treatment. The individual question items and measures contained in this questionnaire are described below.

##### Use of AOD treatment

The criterion variable for this study was AOD treatment utilization. This was assessed by the question: “Have you ever received treatment for AOD problems? “ This item had a “yes” (1) or a “no” (0) response.

##### Need for treatment variables

Two items “Do you think you need AOD treatment?” and “Have others suggested you need AOD treatment” examined internally and externally perceived need for treatment. These items had a “yes” (1) or “no” (0) responses.

The “Stages of Change, Readiness and Treatment Eagerness Scale” (SOCRATES-8D) measured readiness to change AOD use; a situational indicator of perceived need [16]. This 19-item scale consists of three subscales: the seven-item “Problem Recognition” scale, the four-item “Ambivalence” and the eight-item “Taking Steps to Change” subscale. Each item is rated on a 5-point Likert scale, with responses ranging from “strongly disagree” (1) to “strongly agree” (5). The SOCRATES has good construct validity; predicting treatment initiation, treatment engagement and treatment outcomes [16]. Good internal reliability coefficients have been reported, with coefficients ranging from .68 to .89 for the subscales and test-retest reliability correlations ranging from .83 to .99 [16,17]. This study obtained alpha coefficients ranging from .91 to .95 for the subscales.

##### Predisposing factors

The following socio-demographic variables were included in the analysis: age (with responses ranging from 18 to 53), gender (male or female), race (Black African or Coloured), number of years of education received and neighbourhood disadvantage. Number of years of education received was treated as a continuous variable with responses ranging from 0 years to 15 years for those with completed Bachelor degrees.

Neighbourhood disadvantage was assessed using the ten-item “Neighbourhood Environment Scale” (NES) which includes items that examine perceptions of neighbourhood poverty, neighbourhood dilapidation, perceived drug dealing and drug use in the neighbourhood, and perceptions of neighbourhood safety [18]. Items are rated on a five-point Likert scale from “strongly disagree” (1) to “strongly agree” (5). This study obtained a Cronbach alpha coefficient of .82 for the NES.

##### Enabling and restricting factors

A five-item “Affordability barriers scale” [19] measured the extent to which treatment costs, concerns about loss of income due to time taken from work, and transport costs hampered treatment utilization. Items are rated on a five-point Likert scale; with responses ranging from “a very small extent” (1) to “a very large extent” (5). Aggregated responses averaged to give a composite score (ranging from 1 to 5), with higher scores indicating more cost barriers. A Cronbach alpha coefficient of .84 was obtained for this scale.

“Awareness of AOD treatment services” was examined through a single-item question that asks participants to list all known AOD treatment services. Based on responses to this question, the number of known AOD treatment facilities is calculated, with larger numbers indicating greater awareness of services. Responses to this item ranged from 0 known facilities to 8 known services.

“Geographic access to AOD treatment services” was examined through a single-item question that asks participants to estimate the amount of time it took (in 15 minute intervals) to travel to the nearest AOD treatment service. Responses to this item ranged from one 15-minute interval to nine 15-minute intervals (representing 135 minutes), with higher scores on this item reflecting greater geographic access barriers to treatment.

The 10-item “Stigma Consciousness Scale” was used to examine internalized stigma related to participants’ AOD use [20]. More specifically, this scale measures expectations of being judged negatively on the basis of one’s AOD use. Item responses range on 10-point Likert scale from “strongly disagree” (1) to “strongly agree” (10). Responses are summed and averaged to give a composite stigma consciousness score. Possible composite scores range from 1 to 10, with higher scores reflecting more internalised stigma. Initial studies obtained a Cronbach alpha coefficient of .87 for this scale [19]. This study obtained a Cronbach alpha coefficient of .84 for the scale.

The 10-item “Treatment concerns” scale [19] was used to measure individual concerns about what happens in AOD treatment. This scale includes items that explore fears about the kind of treatment that they might receive, fears about what might happen to them during treatment, and fears about the kinds of people they might meet during treatment. Item responses range on five-point Likert scale from “to a very small extent” (1) to “a very large extent” (5). Aggregated responses are averaged to give an overall score (ranging from 1 to 5), with higher scores reflecting greater treatment concerns. This scale has good internal consistency, with a Cronbach alpha coefficient of .90 being obtained for the scale.

### Data analysis

Bivariate comparisons of the utilization variable and the predisposing, enabling and need measures were conducted separately for each race group. Chi-square tests of association were conducted on categorical variables by utilization and odds ratios (OR) were calculated to measure the strength of these associations. Independent sample *t*-tests were used to compare the utilization groups on continuous variables and point-biserial correlation coefficients (*r*_pb_) were used to measure the strength of these associations. Following this, multiple logistic regression analyses were performed to determine which variables were independently associated with treatment use. Utilization was regressed separately for Black African and Coloured participants so that the unique profile of variables associated with utilization could be identified for each group. For each regression analysis, only the predisposing, enabling and need variables that were significantly and moderately associated (OR ≥ 2.5 or *r*_pb_ ≥ 0.25) with utilization in bivariate analyses were entered into the model.

### Results

#### Sample description

The final sample consisted of 434 cases and 555 controls (N = 989). Chi-square tests of association revealed that cases and controls did not differ by gender or race. Similarly, independent sample *t* tests showed that the mean age and level of education did not differ among cases and controls (Table 1).

**Table 1:** **Demographic information for the overall sample** (**N** = **989**)

**Variable**	**Cases% (n)**	**Control% (n)**	**Chi-square/ t -test (p)**	**Overall% (n)**
**Male**	54.4% (236)	50.3% (279)	1.65 (0.20)	52.1% (515)
**Female**	45.6% (198)	49.7% (276)		47.9% (474)
**Black**/**African**	50.9% (221)	50.3% (279)	0.04 (0.84)	50.6% (500)
**Coloured**	49.1% (213)	49.7% (276)		49.4% (489)
**Mean age in years (SD)**^**a**^	24.95 (4.81)	25.43 (5.98)	1.38 (0.17)	25.22 (5.51)
**Mean education** - **grade (SD)**	11.55 (1.57)	11.45 (1.52)	−0.95 (0.34)	11.50 (1.54)
**Total (N)**	434	555	-	989

^a^*SD* Standard deviation.

#### Bivariate analyses

##### Predisposing variables

Years of education was significantly associated with treatment use for Black African (*t* (424) = 2.91; p = 0.004) and Coloured participants (*t* (497) = -0.43; p <0.001). While the number of years of education was negatively correlated with treatment use for Black African participants (*r*_pb_ = -0.13), it was positively associated with treatment use among Coloured participants (*r*_pb_ = 0.19); however the effect sizes of these associations were weak. In addition, age (*t* (487) = 2.37; p = 0.018) and NES (*t* (487) = 5.64; p <0.001) were associated with treatment use for Coloured participants (Table 2). However the effect sizes of these associations were weak (*r*_pb_ = -0.11 for age and *r*_pb_ = -0.25 for neighbourhood disadvantage).

**Table 2:** **Predisposing**, **need and enabling variables by utilization for each race group**

**Variables**	**Coloured**			**Black/African**		
	**No use controls (N = 276) Mean (SD)**	**Treatment use (N = 213) Mean (SD)**	**Effect size** (**OR**^**a**^: **95**% **CI**^**b**^/ ***r***_**pb**_^**c**^	**No use controls (N = 279) Mean (SD)**	**Treatment use (N = 221) Mean (SD)**	**Effect size** (**OR**: **95**% **CI**/ ***r***_**pb**_
***Predisposing variables***						
Age	26.22 (5.95)	24.98 (5.54)	−0.11*	24.63 (5.92)	24.93 (3.99)	0.03
Education (years)	10.96 (1.51)	11.54 (1.47)	0.19**	11.95 (1.37)	11.55 (1.66)	−0.13**
NES	43.77 (3.49)	41.37 (5.86)	−0.25**	40.96 (2.71)	41.47 (4.17)	0.07
***Need for treatment variables***						
Think need treatment (n,%)	128 (46.4%)	133 (62.4%)	1.92: 1.34-2.77	192 (68.8%)	210 (95.0%)	8.65: 4.48-16.69
Others think need treatment (n,%)	195 (70.7%)	190 (89.2%)	3.43:2.07-5.68	199 (71.3%)	202 (91.4%)	4.27:2.50-7.31
SOCRATES- Problem recognition	21.61 (8.07))	26.37 (6.94)	0.30***	22.89 (6.07)	27.86 (3.67)	0.43***
SOCRATES-Ambivalence	12.66 (4.47)	15.06 (3.50)	0.28***	13.95 (3.20)	16.04 (2.04)	0.35***
SOCRATES- Taking steps	17.57 (5.61)	22.86 (7.61)	0.37***	17.30 (3.45)	27.20 (8.28)	0.63***
***Enabling variables***						
Treatment concerns scale	28.36 (9.27)	31.61 (6.05)	0.20***	24.52 (7.29)	27.86 (8.65)	0.21***
Stigma consciousness scale	7.95 (1.51)	8.70 (1.34)	0.25***	7.30 (1.48)	8.48 (1.88)	0.33***
Awareness of AOD Treatment (# of known treatment centres)	2.44 (0.99)	3.83 (0.80)	0.60***	1.65 (0.67)	3.96 (0.86)	0.84***
Geographic access (Time to treatment)	3.63 (0.59)	2.92 (0.80)	−0.45***	3.70 (0.50)	2.53 (0.64)	−0.72***
Affordability barriers	37.71 (6.48)	24.98 (8.19)	−0.66***	39.81 (5.81)	30.73 (9.75)	−0.50***

^a^*OR* Odds Ratio.

^b^ 95% CI = 95% confidence intervals.

^*c*^*r*_pb =_ point-biserial correlation coefficient.

*p < .05; ** *p* < .01; *** *p* < .001.

##### Need variables

The categorical variable “do you think you need AOD treatment” was significantly associated with utilization for both race groups (χ^2^ (1, N = 500) = 53.74, *p* <0.001 for Black African participants; χ^2^ (1, N = 489) = 12.47, *p* <0.001 for Coloured participants). The odds of utilizing treatment were almost nine-fold greater for Black African participants (OR = 8.65; CI (95): 4.48-16.69) and almost two-fold greater for Coloured participants (OR = 1.92; CI (95): 1.34-2.77) who thought they needed AOD treatment compared to participants who did not think they needed treatment. In addition, the categorical variable “Have others suggested you need AOD treatment?” was significantly associated with utilization for both race groups (χ^2^ (1, N = 500) = 31.30, *p* <0.001 for Black African participants; χ^2^ (1, N = 489) = 24.71, *p* <0.001 for Coloured participants). The odds of entering treatment were four-fold greater for Black African participants (OR = 4.27; CI (95): 2.50-7.31) and about three-fold greater for Coloured participants (OR = 3.43; CI (95): 2.07-5.68) for whom others had suggested the need for AOD treatment, relative to participants for whom others had not suggested the need for treatment (Table 2).

Significant differences were found between cases and controls on the SOCRATES-*D scales (Table 2). For both race groups, the treatment use group reported higher scores on the “Problem Recognition” scale (*t* (498) = -10.72, p <0.001 for Black Africans; *t* (481) = -7.01, p <0.001 for Coloureds), “Ambivalence” scale (*t* (498) = -8.44, p <0.001 for Black Africans; *t* (487) = -6.45, p <0.001 for Coloureds) and the “Taking Steps to Change” scale (*t* (498) = -18.09, p <0.001 for Black Africans; *t* (487) = -8.84, p <0.001 for Coloureds) than the treatment non-use group (Table 2). All of these scales were moderately associated with treatment use for Coloured and Black African participants (with *r*_pb_ coefficients ranging from 0.28-0.43), except for the “Taking Steps to Change” scale which was strongly associated (*r*_pb_ = 0.63) with treatment use among Black African participants (Table 2).

##### Enabling/restricting variables

For both race groups, cases obtained significantly higher scores on the “Treatment Concerns” (*t* (430) = -4.58, p <0.001 for Black African; *t* (475) = -4.45, p <0.001 for Coloured participants) and “Stigma consciousness” scales (*t* (498) = -7.83, p <0.001 for Black African; *t* (487) = -7.53, p <0.001 for Coloured participants) than controls. The “Stigma consciousness” scale was moderately associated with utilization for Black African (*r*_pb_ = 0.33) and Coloured participants (*r*_pb_ = 0.25), but the “Treatment Concerns” scale was weakly associated with utilization (*r*_pb_ ranging from 0.20-0.21). Cases reported significantly fewer geographic access barriers, that is shorter travelling times to the nearest treatment centre, (*t* (498) = 7.39, p <0.001 for Black Africans; *t* (487) = 11.24, p <0.001 for Coloureds), fewer affordability barriers (*t* (498) = 12.92, p <0.001 for Black Africans; *t* (395) = 18.62, p <0.001 for Coloureds) and greater awareness of treatment services than controls (*t* (498) = -34.09, p <0.001 for Black Africans; *t* (487) = -16.64, p <0.001 for Coloureds). These variables were strongly associated with utilization for both Black African (*r*_pb_ ranging from 0.50-0.84) and Coloured participants (*r*_pb_ ranging from 0.45-0.66). In addition, Black African participants who had never accessed treatment had significantly higher scores on the “Affordability barriers” scale than their Coloured counterparts (t (553, N = 555) = 4.03, *p* < 0.001).

#### Logistic regression of utilization

Utilization was regressed separately for each race group while controlling for the potential confounding effect of gender. Only variables that were at least moderately associated with utilization during bivariate analysis were entered into the regression models. Variables entered into the regression model for Black African participants included: Think that need treatment; Others suggesting the need for treatment; the SOCRATES Problem Recognition, Ambivalence, and Taking Steps to Change subscales; the Stigma Consciousness scale; Geographic access (travel time to treatment); Awareness of AOD treatment services, and the Affordability barriers scale. Variables entered into the regression model for Coloured participants included: the NES, Others suggesting the need for treatment; the SOCRATES Problem Recognition, Ambivalence, and Taking Steps to Change subscales; the Stigma Consciousness scale; Geographic access (travel time to treatment); Awareness of AOD treatment services, and the Affordability barriers scale. A test of the full model versus the model with the intercept only was statistically significant for Black African participants (χ^2^ (10; N = 500) = 590.91, *p* < 0.001) and Coloured participants (χ^2^ (10; N = 489) = 422.89, *p* < 0.001). These models accounted for approximately 69% (Nagelkerke R^2^ = 0.691) and 58% (Nagelkerke R^2^ = 0.579) of the estimated variance in utilization for Black African and Coloured participants, respectively. According to the Hosmer and Lemeshow test, the models were a good fit for the data (χ^2^ (8; N = 500) = 3.07, *p* = 0.798 for Black African participants; χ^2^ (8; N = 489) = 6.52, *p* = 0.589 for Coloured participants).

There were no predisposing variables significantly associated with utilization after statistically adjusting for the other variables in the models (Table 3). When holding the predisposing and enabling variables constant, Black African participants had several need-for-treatment variables significantly associated with utilization (Table 3). Specifically, the odds of treatment access increased more than 13-fold (OR = 13.70; CI (95): 1.44-125.00) for Black African participants who thought they needed to go for AOD treatment (compared to those who did not think they needed treatment) and more than 38-fold (OR = 38.46; CI (95): 3.98-333.33) for those for whom others had suggested the need for AOD services relative to those who did not receive this advice. In addition the SOCRATES “Taking Steps” scale was significantly associated with access for Black African participants; with a one unit increase in scale scores (reflecting greater readiness to change) increasing the odds of entering AOD treatment by a multiplicative factor of 1.30 (OR = 1.30; CI (95): 1.13-1.48). There were no treatment need variables significantly associated with treatment use among Coloured participants (Table 3).

**Table 3:** **Summary of multiple logistic regression analyses using predisposing**, **enabling and need factors as predictors of substance abuse treatment utilization**

**Independent variables**	**Coloured participants (N = 489)**	**Black African participants (N =500)**
	**OR **^**a **^**(95% CI)**^**b**,**c**^	**OR (95% CI)**^**d**^
**Predisposing variables**		
Gender (Male)	0.66 (0.34-1.31)	0.98 (0.21-4.57)
NES (values range from 10-90)	0.99 (0.91-6.61)	**-----------------**^**e**^
**Need for treatment variables**		
Think need treatment (Yes)	**-----------------**^**e**^	**13**.**70** (**1**.**44**-**125**.**00**)
Others think need treatment (Yes)	1.28 (0.54-3.06)	**38**.**46** (**3**.**98**-**333**.**33**)
SOCRATES Problem Recognition scale (values range from 7 to 35)	1.04 (0.95-1.13)	0.87 (0.73-1.05)
SOCRATES Ambivalence scale (values range from 4 to 20)	1.04 (0.89-1.23)	1.12 (0.87-1.45)
SOCRATES Taking Steps to Change scale (values range from 8 to 40)	1.05 (0.99-1.12)	**1**.**30** (**1**.**13**-**1**.**48**)
**Enabling**/**restricting variables**		
Awareness: # known treatment centres (values range from 0-8)	**4**.**42** (**2**.**96**-**6**.**61**)	**35**.**50** (**11**.**33**-**111**.**25**)
Geographic access: Time to treatment in 15 min intervals (values range from 1-9)	**0**.**49** (**0**.**30**-**0**.**81**)	**0**.**04** (**0**.**01**-**0**.**15**)
Affordability barriers scale (values range from 1-5)	**0**.**83** (**0**.**79**-**0**.**87**)	**0**.**90** (**0**.**83**-**0**.**98**)
Stigma consciousness scale (values range from 1-10)	2.04 (1.52-2.73)	1.31 (0.89-1.95)

^a^*OR* Odds Ratio.

^b^ 95% CI = 95% confidence intervals.

^c^ Model summary: χ^2^ (10; N = 489) = 422.89, *p* < 0.001; Nagelkerke R^2^ = 0.579.

^d^ Model summary: χ^2^ (10; N = 500) = 590.91, *p* < 0.001; Nagelkerke R^2^ = 0.691.

^e^ Variables not entered into the model.

^f^ The bolded odds ratios refer to statistically significant associations.

Several enabling variables were significantly and strongly associated with utilization for Black African and Coloured participants (Table 3). Awareness of services was positively associated with utilization for both groups. For every additional treatment centre that Black African or Coloured participants knew of, the odds of utilizing treatment increased by a multiplicative factor of 35.50 (OR = 35.50; CI (95): 11.33-111.25) and 4.42 (OR = 4.42; CI (95): 2.96-6.61), respectively. Geographic access was also associated with utilization for both races. For every 15 minute increase in travelling time to treatment, the odds of accessing treatment were reduced by 51% for Coloured (OR = 0.49; CI (95): 0.30-0.81) and 96% (OR = 0.04; CI (95): 0.01-0.15) for Black African participants. Affordability barriers also were significant partial predictors of utilization for both races, with every one unit increase in the affordability barriers scale reducing the odds of accessing treatment by 17% for Coloured (OR = 0.83; CI (95): 0.79-0.87) and 10% (OR = 0.90; CI (95): 0.83-0.98) for Black African participants.

One other enabling variable, stigma consciousness, was significantly associated with treatment utilization for Coloured participants only. Among Coloured participants, every one unit increase in the Stigma Consciousness scale doubled the odds of accessing treatment (OR = 2.04; CI (95): 1.52-2.73; Table 3).

## Discussion

Previous studies have identified racial disparities in the use of AOD treatment services in South Africa, with Black African and Coloured persons consistently under-represented in speciality AOD treatment services [5,21]. However there has been a paucity of research examining the factors that underpin these racial disparities. To the best of our knowledge, this study is the first to examine the unique profile of barriers to AOD treatment use among Black African and Coloured persons. As such, findings from this study potentially deepen current understandings of how racial disparities in access to AOD treatment can be ameliorated and service coverage expanded to include these underserved populations. More specifically, the study found several similarities and differences in terms of the profile of treatment barriers experienced by Black African and Coloured AOD-using persons.

First, findings suggest that key structural barriers to accessing AOD treatment are common among people disadvantaged during the apartheid regime, irrespective of race group membership. More specifically, geographic access barriers (related to length of time taken to travel to the nearest treatment facility) and affordability barriers were significantly associated with not accessing AOD treatment for both Black African and Coloured participants. These findings are not altogether surprising given that several studies have noted similar structural barriers to accessing public health services in post-apartheid South Africa for Black African and Coloured South Africans [6,8,22]. Although these key barriers were reported by Black African and Coloured persons, variations in the extent to which these barriers impact on the probability of AOD treatment use were noted.

Specifically Black African persons appear more susceptible to the effects of geographic access barriers than Coloured persons. This may be the result of the enduring spatial inequalities that exist between race groups in the country. During apartheid both Black African and Coloured South Africans were forced to reside in areas that were geographically removed from urban hubs, however apartheid planning ensured that Black African communities were relatively further removed from these well-resourced urban centres and had relatively less infrastructure than Coloured communities [6,8]. This increased the distance and time required to travel to the nearest treatment facility. Unfortunately, this spatial segregation did not stop with the end of apartheid, with post-apartheid low-cost housing developments for Black African communities who previously had little access to social housing still located far from the urban periphery [23]. In addition, these areas are poorly served by public transport, with Black African populations having significantly less access to public transport services than Coloured populations [23]. Together these factors most likely underpin Black African persons’ increased vulnerability to geographic access barriers through increasing the distance, time and difficulty in travelling to the nearest AOD treatment facility.

These findings suggest several strategies for improving access to AOD treatment for Black African and Coloured persons (although Black African persons may benefit most from interventions to reduce geographic barriers to treatment entry). First, careful consideration should be given to the positioning of new AOD treatment services and how these services are delivered. If new services are located far from Black African communities they will entrench geographic access barriers for this population. In addition to building new facilities close to underserved communities, another strategy would be to introduce mobile outpatient AOD services into underserved communities. Not only would mobile services improve treatment availability, but they would reduce Black African persons’ travel time (and concomitant costs) to the nearest AOD service. As mobile services have never been used for the delivery of AOD services in South Africa, future research should consider piloting a mobile AOD service to test whether this mode of service delivery is feasible to implement and acceptable to the target population.

In addition, findings suggest that affordability barriers are somewhat stronger determinants of AOD treatment access for poor Coloured persons compared to their Black African counterparts. This finding is counterintuitive given evidence that Black African communities experience more financial barriers to accessing health services than Coloured communities [9,23]. This surprising finding does not mean that affordability barriers are not significant determinants of AOD treatment access for Black African persons. A closer examination of the data shows that Black African persons who had never accessed treatment reported significantly more affordability concerns compared to their Coloured counterparts. It is possible that the relatively homogenous responses of Black African participants on the affordability barriers scale compared to Coloured participants may have reduced the possibility of detecting any measurable association between these barrier variables and access for this population subgroup in multivariate analyses. Regardless of the reason for these differences, findings point to the importance of reducing treatment costs as a means to improve AOD treatment use for both Black African and Coloured communities. In South Africa, AOD treatment services are only offered by stand-alone treatment facilities. There are few free AOD treatment services available and most not-for -profit services require user co-payment fees [21]. One strategy for expanding the availability of free AOD services to poor South African communities would be to initiate AOD intervention services within the country’s free primary health care and social service systems. As these primary health and social services are located within easy reach of underserved communities, the provision of AOD services within these settings would alleviate the financial burden of user co-payment fees while also reducing geographic barriers to AOD treatment access.

A shared facilitator to AOD treatment entry for Black African and Coloured persons is awareness of where to go for AOD treatment, with the likelihood of AOD treatment use improving with every increase in the number of known treatment facilities. This suggests that access for underserved groups could be improved by increasing public awareness of where and how to access AOD treatment. However, we found differences in the degree to which Black African and Coloured persons were vulnerable to the influence of this variable, with Black Africans relatively more susceptible to the effects of awareness on access than Coloured persons. This could be because of poor health literacy around AOD-related problems and addiction in Black African communities relative to Coloured communities. In recent years, Coloured communities have been the target of several AOD awareness campaigns whereas Black African communities have been relatively neglected due to the perceived low prevalence of AOD problems in these communities [24].

One promising avenue for improving awareness of AOD services (and subsequently treatment use) among Black African communities lies in the important role that relationships with others play in Black African communities. Black African communities have a collectivist cultural orientation that emphasises relatedness to and interdependence with others [25,26]. In comparison, Coloured communities are not as collectivist in cultural orientation and place less emphasis on relatedness to others [25]. Within Black African communities, this emphasis on social and community relatedness impacts on the use of health services; with evidence of social networks in these communities buffering people against the effects of limited awareness and poor health literacy on service use [26,27]. Awareness-related interventions targeted at the level of the social network thus may be an effective strategy for improving access to AOD treatment in Black African communities. This study’s finding of considerably greater odds of accessing treatment among Black African participants for whom significant others had suggested the need for AOD treatment (compared to those for whom significant others had not) provides support for the potential role that social networks can play in facilitating treatment access in Black African communities. Community health workers (that is health workers without formal health care training who function to promote community health, provide preventive services, and address barriers to health care [28]) are ideally placed to conduct community outreach to improve awareness of AOD treatment and to provide support for families and social networks dealing with AOD problems. With a little investment in training, this cadre of health worker could also help identify and encourage people with AOD problems to seek services.

Further distinctions in the profile of factors associated with AOD treatment use among Black African and Coloured persons were found. Perceived need for treatment (as assessed by the question do you think you need treatment) and the SOCRATES “taking steps to change” scale were associated with AOD treatment entry for Black African participants only. These findings show that Black African participants are most likely to enter treatment when they have a strong perceived need for treatment and are already taking steps to change their AOD use. In contrast, these variables were not associated with access to AOD treatment for Coloured participants, suggesting that Coloured persons are able to access treatment regardless of their degree of problem recognition or readiness to change. These findings imply that Black African persons enter AOD treatment at a later point than Coloured persons, when their AOD problems are apparent and they identify the importance of change. This may be a result of greater difficulties in accessing AOD treatment relative to their Coloured counterparts.

A further variation between Black African and Coloured AOD treatment seekers was that perceived stigma was significantly (and positively) associated with AOD treatment use among Coloured participants only. This finding of a positive association between stigma and treatment use is surprising given previous research which notes that stigma hinders rather than promotes entry into AOD treatment [29]. One possible explanation for this unexpected finding lies in this study’s measurement of perceived stigma. This study employed the stigma consciousness scale which measures perceptions of being judged negatively on the basis of one’s AOD use rather than on the basis of one’s use of AOD treatment services [20]. It is quite possible that high levels of stigma around problematic AOD use cause such distress that people enter treatment partly to alleviate this distress. Earlier qualitative research which reported that people from disadvantaged communities experience stigma in relation to their problematic AOD use rather than their use of AOD services [30] provides some support for this explanation. Further, this qualitative research pointed to the intense stigma associated with the problematic use of methamphetamine. As methamphetamine-related problems are significantly more prevalent among Coloured relative to Black African communities [5], this may help explain why perceived stigma was such a salient facilitator of AOD treatment entry for Coloured participants but not for Black African participants.

Findings from this study should be considered in the light of several limitations. As a case-control design precludes a temporal examination of the factors associated with treatment utilization, inferences about causality cannot be made. Further, the use of a matched design ruled out an examination of race differences in the likelihood of AOD treatment use. Third, the crude conceptualisation of access employed by this study prevented an examination of whether there were differences between participants who had unsuccessfully attempted and those who had never attempted to access services. Fourth, strict selection criteria may have reduced the variability of the sample and impacted on the extent to which many predisposing variables were associated with treatment entry. Finally, as this study was limited to adults from disadvantaged communities in Cape Town, the extent to which findings are representative of more rural or other urban regions in South Africa is questionable. Yet as the Western Cape is one of the best resourced provinces in terms of AOD services [21], it is highly likely that these barriers are even more salient elsewhere in the country.

These limitations highlight the need for further research on AOD treatment use in South Africa. Future research should include longitudinal prospective studies that track people with AOD-related difficulties and allow researchers to unpack the factors that precipitate entry into AOD treatment utilization for each race group. These longitudinal studies will also allow researchers to monitor racial disparities in accessing treatment and evaluate the impact of interventions to reduce these disparities. To address concerns about the external validity of findings, studies on factors associated with AOD treatment use in other parts of the country (particularly rural regions) and for other population subgroups (such as adolescents) are required. In addition, researchers should conduct experimental intervention studies that test whether study recommendations for improving access to AOD treatment among poor Black African and Coloured South Africans impact positively on AOD treatment use.

## Conclusion

Despite some limitations this study provides evidence of differences in barriers to AOD treatment use among poor Black African and Coloured South Africans; with Black Africans appearing more vulnerable to the effects of geographic access and awareness barriers and entering treatment at a later point than their Coloured counterparts. Findings suggest several strategies for improving access for these underserved groups. First, to avoid entrenching geographic barriers, new AOD services should be placed in locations easily accessible by public transport and in communities with high service needs and poor service coverage. Second, mobile AOD services may offer an efficient solution to affordability and geographic access barriers by reducing the infrastructure costs of new facilities and allowing services to be moved between and within communities. Third, if AOD intervention services are provided within the primary health and social service system this would reduce affordability and geographic access barriers while expanding the availability of care. Finally, service providers should consider using community health workers to conduct outreach in order to improve awareness of AOD treatment within Black African communities. Through increasing community awareness of when, where and how to access services, outreach workers can positively impact on Black African South Africans’ use of AOD treatment services.

## Abbreviations

AOD: Alcohol and other drug.

## Competing interests

The author declares she has no competing interests.

## Author’s contribution

BM was the principal investigator for the study and led all aspects of the study design, analysis and write up of the results.

## Pre-publication history

The pre-publication history for this paper can be accessed here:

http://www.biomedcentral.com/1472-6963/13/177/prepub
